# Partial Failure of Milk Pasteurization as a Risk for the Transmission of *Campylobacter* From Cattle to Humans

**DOI:** 10.1093/cid/civ431

**Published:** 2015-06-10

**Authors:** Anand M. Fernandes, Sooria Balasegaram, Caroline Willis, Helen M. L. Wimalarathna, Martin C. Maiden, Noel D. McCarthy

**Affiliations:** 1 Operations Directorate; 2 Field Epidemiology Services; 3 Food, Water and Environment Laboratory, Public Health England; 4 Department of Zoology; 5 National Institute for Health Research Health Protection Research Unit in Gastrointestinal Infections, University of Oxford; 6 Warwick Medical School, University of Warwick, United Kingdom

**Keywords:** *Campylobacter*, cattle, milk, whole-genome sequencing, pasteurization

## Abstract

***Background.*** Cattle are the second most common source of human campylobacteriosis. However, routes to account for this scale of transmission have not been identified. In contrast to chicken, red meat is not heavily contaminated at point of sale. Although effective pasteurization prevents milk-borne infection, apparently sporadic infections may include undetected outbreaks from raw or perhaps incompletely pasteurized milk.

***Methods.*** A rise in *Campylobacter* gastroenteritis in an isolated population was investigated using whole-genome sequencing (WGS), an epidemiological study, and environmental investigations.

***Results.*** A single strain was identified in 20 cases, clearly distinguishable from other local strains and a reference population by WGS. A case-case analysis showed association of infection with the outbreak strain and milk from a single dairy (odds ratio, 8; Fisher exact test *P* value = .023). Despite temperature records indicating effective pasteurization, mechanical faults likely to lead to incomplete pasteurization of part of the milk were identified by further testing and examination of internal components of dairy equipment.

***Conclusions.*** Here, milk distribution concentrated on a small area, including school-aged children with low background incidence of campylobacteriosis, facilitated outbreak identification. Low-level contamination of widely distributed milk would not produce as detectable an outbreak signal. Such hidden outbreaks may contribute to the substantial burden of apparently sporadic *Campylobacter* from cattle where transmission routes are not certain. The effective discrimination of outbreak isolates from a reference population using WGS shows that integrating these data and approaches into surveillance could support the detection as well as investigation of such outbreaks.

## Abstract (precis)

An outbreak investigation identified a plausible transmission route that may contribute to the large and poorly characterized human disease burden of *Campylobacter jejuni* from cattle and demonstrated an approach to testing this hypothesis through integration of genomic analysis in surveillance.

**(See the Editorial Commentary by Osterholm on pages 910–1.)**

*Campylobacter* is the commonest cause of bacterial gastroenteritis in humans, with chicken and cattle the first and second most common sources, respectively [1–3]. Population genetic models have attributed 39% [2] and 18%–38% [1] of human infection in the United Kingdom to ruminant (cattle or sheep) sources, and 20%–30% in New Zealand [3], with cattle identified as the main ruminant source. High prevalence and concentration of *Campylobacter* on chicken contrasts with low prevalence and concentration on red meat at retail [4, 5], and transmission routes to humans from ruminants are unclear despite the ruminant-associated burden of well over 100 000 cases per year in England alone [2, 6].

Although detected *Campylobacter* outbreaks in England have included some from raw milk sources [7, 8], especially locally distributed, this pattern has changed with near-universal pasteurization of milk. Identified outbreaks are now mostly associated with foods containing chicken liver prepared by the catering industry [9]. In the United States, the decline has been less marked; 1 ruminant-associated *Campylobacter jejuni* subtype has been found in outbreaks linked to unpasteurized milk, in addition to 56 apparently sporadic cases where source of infection was generally unknown [10]. Among apparently sporadic cases of *Campylobacter* in Minnesota, 6% (407) reported consuming raw milk compared with 2.3% among the general population. Raw milk consumption was estimated by extrapolation to have caused >12 000 cases in this population from 2001 to 2010 [11]. The occurrence of so many raw milk–associated cases in Minnesota, in the absence of detected outbreaks, may be a feature of the difficulty of detecting outbreaks for a widely distributed food [12], especially if outbreaks are small and the distribution diffuse [7, 8, 13]. More generally, *Campylobacter* outbreaks have mainly been identified in socially or geographically defined groups [7–9], despite a biology of persistence but not growth on foods [14, 15], so that distributed outbreaks might be expected to be more common.

The combination of a large burden of human campylobacteriosis originating from cattle, unexplained transmission routes from this source, and evidence that a large burden of apparently sporadic unpasteurized milk–associated disease can occur without detected outbreaks [11] raises the question of whether imperfectly pasteurized milk might also cause undetected outbreaks that contribute to human campylobacteriosis. If pasteurization in large-volume, widely distributed supplies reduces risk substantially, but sometimes incompletely (eg, due to partial failures), the resulting low-level distributed contamination would be likely to produce even more diffuse outbreaks than with raw milk. Current human disease surveillance would not provide a robust form of monitoring to detect these outbreaks.

When bacterial subtyping has been applied to milk-borne *Campylobacter* outbreaks, a single or dominant subtype has usually been identified [10, 16–19], including 1 recent family farm outbreak where isolates from family members, cattle feces, and milk tanks were shown to be almost identical [20] by whole-genome sequence (WGS) multilocus sequence typing (wgMLST) [21, 22]. This shows that milk-borne outbreaks are at least sometimes due to a single strain. Preliminary work suggests that WGS analysis can detect distributed *Campylobacter* outbreaks [21], even though preceding molecular methods have not been effective for detecting outbreaks of this diverse pathogen [23, 24].

Here we describe an outbreak due to unidentified inadequate pasteurization at a dairy supplying a local population. Localized exposure of an age group in which this infection is rare allowed detection of a cluster likely to have been missed if distribution were across a wider population. Identification of the source of infection involved integration of WGS data, other epidemiological data, and environmental investigation showing the benefits of triangulating WGS data with more familiar forms of epidemiological information [25]. We also used this example to test whether integration of genome sequencing in surveillance could detect epidemiologically related cases occurring in a less demographically distinct group.

## METHODS

### Epidemiological Methods

#### Case Definition

The outbreak control team case definition included any laboratory-confirmed case of *Campylobacter* gastroenteritis at a laboratory serving the population of a small island, identified during October 2011, with onset after 29 September, without preceding foreign travel, and where the isolate was sensitive to ciprofloxacin and erythromycin.

Subsequently, an outbreak strain case definition to allow case-case analysis used the criteria of allele differences at ≤12 of the genetic loci used in *Campylobacter* wgMLST analysis [21]. Cases in households where another case had occurred ≥3 days earlier were considered as secondary to allow repeat analysis excluding probable and possible secondary cases.

#### Case Information

A standard questionnaire for gastrointestinal illness applied by the local public health authorities gathered information on symptoms, onset time, and food and other exposures in the week preceding onset. This was later modified to examine milk consumption in more detail. Cases, or parents of child cases, were interviewed in person or by telephone. Information obtained was recorded on the case management system used by English public health authorities.

#### Microbiological Methods for Human Samples

Stool specimens submitted to the hospital laboratory serving the island population were cultured for *Campylobacter* species using British standard methods. Once an outbreak was suspected, available isolates, which are usually discarded, were retained for genome sequencing. DNA was extracted and sequencing performed on the Illumina Hi-Seq platform as described elsewhere [21].

#### Food Chain Investigation

The milk supply chain for schools was later investigated given the concentration of infection among school-aged children and frequent reports by these cases of school milk consumption.

#### Food Microbiology and Biochemistry

Milk samples were taken from the dairy milk tank and after bottling and analyzed at the Food Water and Environmental Microbiology Laboratory, Porton Down, for alkaline phosphatase (according to International Organization for Standardization 11816–1:2006) to test for adequacy of pasteurization [26], Enterobacteriaceae using the TEMPO automated most probable number technique [27], and for the presence of *Campylobacter* using Health Protection Agency Standard Procedure F21.

#### Bioinformatics Analysis

The 23 available isolates from the laboratory catchment population were analyzed with a reference population comprising 65 contemporaneous isolates from an ongoing genomic surveillance project in Oxfordshire, United Kingdom (isolates cultured between 29 September and 22 October) given evidence for seasonal but limited geographical variation in *Campylobacter* subtypes in England [28–30]. The Genome Comparator tool was used within BIGSdb [31] to perform wgMLST analysis [22] using the 1643 genetic loci validated for this form of analysis [21]. Pairwise differences were estimated among outbreak and reference population isolates to discriminate clusters against a background population.

Following the identification of an outbreak strain as described above, a more challenging discriminatory task was set, to separate outbreak isolates from a reference population with the same standard 7-locus MLST [32] as the outbreak strain (ST21). This used all ST21 isolates from Oxfordshire isolated during September, October, and November (n = 29) as a surrogate to test the capacity of genome sequencing to distinguish the outbreak cluster from a wider background population. This evaluation compared the distributions of pairwise differences between (1) each pair of outbreak strain isolates, (2) each pair of reference population isolates, and (3) each outbreak strain isolate and each reference isolate. Genome sequences for all isolates used in the analyses are accessible on the pubMLST/Campylobacter database.

#### Case-Case Analysis

Following confirmation of an outbreak strain, a case-case analysis [33] was undertaken comparing outbreak strain cases with all other isolates from the island population that had different genome sequences or had not been genome sequenced, from people without a history of foreign travel. Odds ratios (ORs) and 95% confidence intervals (CIs) were estimated by logistic regression and exact logistic regression implemented in Stata 12, and statistical significance was tested using Fisher exact test.

## RESULTS

### Descriptive Epidemiology

Forty-eight *Campylobacter*-positive samples were reported by the hospital laboratory in October vs an average of 15 during October over the previous 4 years. Eleven were excluded as cases due to symptom onset prior to 29 September (n = 6), foreign travel (n = 2), or antibiotic resistance (n = 3).

**Figure 1.**
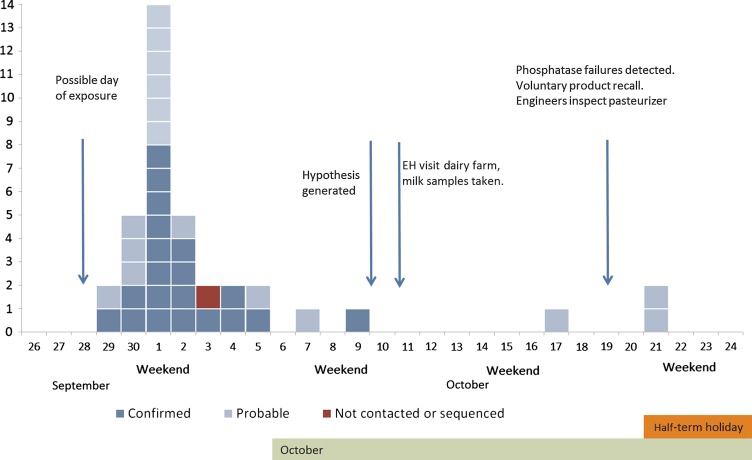
Epidemic curve of cases of *Campylobacter* per day identifying those meeting the original outbreak case definition (probable) and the outbreak strain case definition (confirmed). Abbreviation: EH, environmental health.

Twenty-nine of the 37 cases were in the primary and preschool age range of 1–11 years (78%). The other 8 were aged ≥24 years. Onset dates were mainly (32 of 37) between 29 September and 5 October, peaking on Saturday, 1 October (Figure 1). Cases in schoolchildren were distributed unevenly among 12 schools. Two schools accounted for 52% (15) of cases in the primary and preschool age range.

Diarrhea was reported by all 33 cases for whom symptom information was available, with abdominal pain reported by 88%, fever 70%, and bloody diarrhea 52%. Two children were hospitalized for 1 day each.

### Human Sample Microbiology

Twenty-three isolates were available for genome sequencing. Complete data were available at 1319 genetic loci for all of these and the reference population isolates. Genes were missing or alleles incomplete in 1 or more isolates at an additional 324 loci. Some allelic variation among isolates was evident at all 1319 loci with complete data. A cluster of 20 of the 23 isolates in the outbreak catchment area were almost identical, with differences of ≤4 (mean, 1.3) on all pairwise comparisons. The other 3 isolates from the area were not similar to this cluster, each differing at ≥1132 loci from all members of the cluster and from each other (mean, 1190). Extraction of 7-locus MLST [32] showed that the cluster of 20 isolates shared the 7-locus ST21 genotype and the other 3 were 1 each of ST42, ST257, and ST353. The 65 reference population isolates differed at ≥56 loci from each strain in the outbreak cluster.

**Figure 2.**
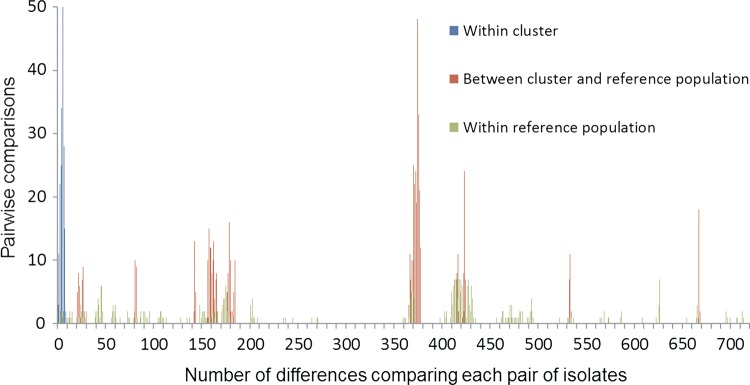
Distribution of pairwise differences between ST21 isolates in an identified cluster of 20 isolates (blue), between isolates in this cluster and a geographically separate but temporally similar reference population of ST21 isolates (red), and within the reference population (green) across 1577 loci.

Analysis comparing the cluster of 20 against the related ST21 reference population showed 1577 shared loci. Identical alleles were present at 602 in all reference and outbreak isolates, whereas 975 showed allelic variation. The pattern of pairwise differences among each population and between the 2 is shown in Figure 2. The cluster of 20 isolates showed a range of 0–8 and mean of 4 pairwise differences ( “within-cluster” differences); comparing each isolate in the cluster with each in the reference population showed differences at between 20 and 668 loci, with a mean of 295 (Figure 2).

### Food Chain Tracing

One dairy supplied milk to all local authority primary schools in the area, to nurseries offering government-funded milk to children <5 years old, and to a small proportion of the wider market including hotels, care homes, and household doorstep deliveries of milk.

### Dairy Inspection and Milk Testing

Temperature thermographs on pasteurization tanks did not identify pasteurization process failure. No basis for postpasteurization contamination was identifiable on review of the packaging process and bottle storage. *Campylobacter* was not isolated from the milk samples taken from the dairy. Seventeen of 22 samples (Table 1) exceeded the alkaline phosphatase level of 350 mU/L specified in European legislation (Commission Regulation [EC] 1664/2006), indicating either failed pasteurization or contamination with raw milk after pasteurization. Enterobacteriaceae counts exceeded the criteria specified for pasteurized milk in European microbiological criteria (EC 2073/2005 as amended in EC 365/2010).

**Table 1.** Phosphatase Test Results on Milk Samples Before and After Repair of the Pasteurizer

Date of Sample	Type of Sample and Alkaline Phosphatase Result^a^, mU/L	
	Whole Milk	Skimmed Milk
10 October	Bottle: 24	
17 October	Tank: **517**	Tank: **627**
	First bottle on line: **578**	First bottle on line: 337
	Last bottle on line: 65	
19 October	Tank: **722**	Tank: **627**
	Bottles: 225, **365**, 250	Bottles: **625, 639, 639**
20 October	Bottles: **906, 880, 837, 903**	Bottles: **608, 601, 632, 539**
Pasteurizer repaired overnight 20–21 October		
21 October	Bottles: 20, 20, 16	Bottles: <10, <10
22 October	Bottles: 22, 16, 18, 12	Bottles: <10, 12, 12, <10, <10
27 October	Bottles: 25, 25, 29, 25, 44	Bottles: 22, 54, 42, 27

^a^ Counts exceeding regulatory standards (350 mU/L) are shown in bold.

Inspection of the pasteurizer by an engineer, in the light of milk-test results, identified problems with heat exchanger plates, rubber gaskets, and an internal control mechanism on a steam control valve. These could have led to the failure of pasteurization for some of the milk passing through the pasteurizer, although most may have reached pasteurization temperatures. The regeneration heat exchanger and hot water set plates were replaced, and the pasteurizer was recalibrated. Subsequent phosphatase tests on 23 samples were within normal limits (Table 1).

### Reported Risk Factors and Association With the Outbreak Strain

Of the 29 cases aged 0–11 years, 27 interviews were completed, including 23 school-aged children. Twenty-one parents of school-aged cases reported school milk consumption by their children in the week before illness. Consumption of school lunches was reported by 17 cases and participation in school swimming by 8 cases. Case-case analysis of the association between milk consumption and illness is summarized in Table 2 (OR, 8.0 [95% CI, 1.4–45.8]). Excluding 5 cases that are considered likely to be household secondary infections, all cases for whom an exposure history was available had consumed milk (OR, 11.7 estimated by exact logistic regression).

**Table 2.** Association Between Illness With the Confirmed Outbreak Strain and Milk Consumption From the Implicated Dairy

Cases	Milk	No Milk	OR	95% CI	*P* Value
Analysis of all cases with available data^a^					
Confirmed case	18	2	8.0	1.4–45.8	.023
Not confirmed	9	8			
Excluding probable secondary cases^b^					
Confirmed case	18	0	11.7	1.4–undefined	.010
Not confirmed	9	5			

Abbreviations: CI, confidence interval; OR, odds ratio.

^a^ Three individuals were not interviewed or did not give information on exposure to milk.

^b^ Five cases occurred in family members of cases and may have been secondary cases.

## DISCUSSION

The distribution of milk from a dairy with pasteurization failures to an insular community, served by a single microbiology laboratory, and including school-aged populations, supported the detection of an epidemiological signal for this outbreak. A combination of descriptive epidemiology, genomic epidemiology, and environmental investigation identified the likely source of infection. Combination of exposure histories and WGS data allowed testing of the hypothesis generated using case-case analysis [33]. Although no single analysis or form of data was conclusive, the combination allows relatively firm inference on the source and process issues that led to human infection. Misclassification of cases with unavailable isolates as controls might have weakened the observed associations but would not have created a false-positive association. A cohort or case-control study would have been useful in confirming this inference and testing for possible effects of confounding that was not possible in our small case-case analysis, particularly if evidence was needed to support enforcement. The main peak of the outbreak was short-lived, which was compatible with infection from a single day's delivery of milk. Additionally, illness did not appear to be evenly distributed across school attendees and other populations exposed to milk from the dairy. This suggests that contamination may have affected only a portion of the milk from the dairy, and for only a limited time. Later tests on dairy milk were negative for *Campylobacter* although showing biochemical evidence for pasteurization failure.

Most reported *Campylobacter* outbreaks are small and detected in defined communities. Some have been larger, with 1 outbreak associated with pasteurized milk believed to have been contaminated after pasteurization, causing an estimated 1644 cases among prisoners in California, but this was nonetheless in a defined group [17]. Noninstitutional outbreaks linked to pasteurization failures have been described where a community distribution was relatively local [34, 35] or where the outbreak was very large (affecting 3500 individuals) and mainly concentrated in schoolchildren [16, 36]. In some incidents, increased awareness of risk due to identified pasteurization failures may have contributed to outbreak detection [34, 35] and, as in the present outbreak, interventions may have contributed to a short duration or the avoidance of recurrence. Reports of recurrent pasteurization failures but only single, time-limited outbreaks [34, 35] fit with our later milk samples testing negative for *Campylobacter* even though phosphatase tests suggested incomplete pasteurization of at least some milk. Contamination of raw milk with *Campylobacter* appears to be uncommon and mainly associated with fecal contamination [19, 37, 38].

Neither the outbreak that we report nor other literature describes the type of diffuse outbreaks that might be anticipated from low-level *Campylobacter* contamination of milk given that it is a product that is typically processed in bulk and widely distributed, and that this pathogen can survive in refrigerated milk for 3 weeks [39] but could not grow [14, 15] in these conditions. It may be that modern large-scale production of pasteurized milk provides complete protection, or that the protection is sometimes <100% but that wide distribution networks for milk make it difficult to detect outbreaks following minor levels of contamination and partial pasteurization failure so that they are lost into the background of apparently sporadic cases. The large burden of unexplained cattle origin human infection highlights the importance of obtaining evidence to identify which of these explanations is correct.

Critically, this investigation and our past work [21] show that the integration of WGS into the surveillance of *Campylobacter* infection will allow the detection of single-strain outbreaks, or those where a single strain is dominant. The 20 outbreak isolates showed allele differences at ≤8 loci on pairwise comparison, with a mean of 4 locus differences, across 1577 loci analyzed. These levels of difference are equivalent to the differences seen between 2 isolates from the same patient and do not appear to occur among isolates with no epidemiological relationship [21]. As a single or dominant subtype has typically been reported for milk-borne *Campylobacter* outbreaks when subtyping has been undertaken [10, 16–20], these outbreaks may be particularly detectable by genomic approaches. This contrasts with outbreaks due to poultry liver–containing foods that often contain multiple strains [40, 41]. Taken together, this suggests that the integration of these data and techniques into routine surveillance of *Campylobacter* could detect diffusely distributed outbreaks that do not produce an epidemiological signal in time and space and which are currently likely to be missed, as well as supporting their further investigation. The investigation of these outbreaks may allow identification of the pathways of the extensive human infection that comes from the cattle *Campylobacter* reservoir and support control measures against this large burden of disease. However, such detection of multiple, relatively small, epidemiologically related clusters may be a double-edged sword. On one hand, small, well-investigated clusters can provide insight into overall risk factors for infectious disease to support control [42]; on the other hand, the difficulties of identifying sources in small outbreaks, especially if cases are diffusely distributed, will limit the practicality or utility of investigating all such clusters. Identifying which leads to follow may be critical to our effective application of these novel technologies.
